# Characterizing Chronic Pain Episodes in Clinical Text at Two Health Care Systems: Comprehensive Annotation and Corpus Analysis

**DOI:** 10.2196/18659

**Published:** 2020-11-16

**Authors:** Luke A Carlson, Molly M Jeffery, Sunyang Fu, Huan He, Rozalina G McCoy, Yanshan Wang, William Michael Hooten, Jennifer St Sauver, Hongfang Liu, Jungwei Fan

**Affiliations:** 1 Division of Digital Health Sciences Department of Health Sciences Research Mayo Clinic Rochester, MN United States; 2 Division of Health Care Policy and Research Department of Health Sciences Research Mayo Clinic Rochester, MN United States; 3 Division of Community Internal Medicine Department of Medicine Mayo Clinic Rochester, MN United States; 4 Division of Pain Medicine Department of Anesthesiology and Perioperative Medicine Mayo Clinic Rochester, MN United States; 5 Division of Epidemiology Department of Health Sciences Research Mayo Clinic Rochester, MN United States

**Keywords:** chronic pain, guideline development, knowledge representation, corpus annotation, content analysis

## Abstract

**Background:**

Chronic pain affects more than 20% of adults in the United States and is associated with substantial physical, mental, and social burden. Clinical text contains rich information about chronic pain, but no systematic appraisal has been performed to assess the electronic health record (EHR) narratives for these patients. A formal content analysis of the unstructured EHR data can inform clinical practice and research in chronic pain.

**Objective:**

We characterized individual episodes of chronic pain by annotating and analyzing EHR notes for a stratified cohort of adults with known chronic pain.

**Methods:**

We used the Rochester Epidemiology Project infrastructure to screen all residents of Olmsted County, Minnesota, for evidence of chronic pain, between January 1, 2005, and September 30, 2015. Diagnosis codes were used to assemble a cohort of 6586 chronic pain patients; people with cancer were excluded. The records of an age- and sex-stratified random sample of 62 patients from the cohort were annotated using an iteratively developed guideline. The annotated concepts included date, location, severity, causes, effects on quality of life, diagnostic procedures, medications, and other treatment modalities.

**Results:**

A total of 94 chronic pain episodes from 62 distinct patients were identified by reviewing 3272 clinical notes. Documentation was written by clinicians across a wide spectrum of specialties. Most patients (40/62, 65%) had 1 pain episode during the study period. Interannotator agreement ranged from 0.78 to 1.00 across the annotated concepts. Some pain-related concepts (eg, body location) had 100% (94/94) coverage among all the episodes, while others had moderate coverage (eg, effects on quality of life) (55/94, 59%). Back pain and leg pain were the most common types of chronic pain in the annotated cohort. Musculoskeletal issues like arthritis were annotated as the most common causes. Opioids were the most commonly captured medication, while physical and occupational therapies were the most common nonpharmacological treatments.

**Conclusions:**

We systematically annotated chronic pain episodes in clinical text. The rich content analysis results revealed complexity of the chronic pain episodes and of their management, as well as the challenges in extracting pertinent information, even for humans. Despite the pilot study nature of the work, the annotation guideline and corpus should be able to serve as informative references for other institutions with shared interest in chronic pain research using EHRs.

## Introduction

### Significance

Chronic pain (ie, pain persisting for >90 days) can be debilitating to both physical and emotional well-being and has resulted in significant socioeconomic costs [1,2]. In 2016, it was estimated that 20.4% (50.0 million) of US adults had chronic pain, with 8.0% (19.6 million) experiencing high-impact chronic pain that can frequently limit life or work activities [1]. The annual costs of medical treatment, lost productivity, and disability programs have been estimated at US $560 billion in the United States alone [3]. Effective treatment and management of chronic pain is difficult, due to complex and frequently multifactorial etiology, intertwined mental health conditions, and progression of pain from a symptom to a disease in itself [4]. Research is urgently needed to understand chronic pain through real-world data and to inform best practices for patient care.

Electronic health records (EHRs) hold great promise for chronic pain research, offering rich and contextualized practice-generated evidence. EHR data may also facilitate examination of the effectiveness and safety of chronic pain interventions [5], which heretofore has been limited. Unstructured narratives (ie, clinical notes) in EHRs are indispensable to understanding the full context of a patient’s experience, and most clinicians prefer the expressiveness of free text in documenting pain [6]. However, there has been no systematic appraisal of EHR narratives surrounding chronic pain. Therefore, as a foundational step toward filling this gap, we annotated and analyzed the clinical notes of patients with chronic pain diagnoses.

Guided by clinical practice and research needs, we annotated information related to body location, severity, causes, social and emotional effects, and interventions associated with chronic pain across a wide range of symptomatology and etiology. We centered around individual episodes of chronic pain, examining notes spanning the period from 6 months before to 2 years after the first chronic pain diagnosis for each patient. A total of 3272 notes were reviewed, and 94 episodes from 62 distinct patients were identified. The results showed that the clinical notes contain valuable information on chronic pain, effectively covering key aspects such as location and cause of pain in more than 90% of the episodes. Moreover, aspects of chronic pain generally not available in structured EHR data, like alternative regimens and the effects on quality of life, also had a sizable presence in the annotated corpus.

### Background

Previous work pointed out that chronic pain surveillance can be limited without using information in clinical text [7], and there has been substantial interest in detecting pain phenotypes based on EHR documents. Heintzelman et al [8] used a proprietary, rule-based system to identify mentions of pain and attributes such as location and severity for 33 prostate cancer patients. Two prior studies developed and analyzed clinical corpora on pain and pain management. Dorflinger et al [9] sampled 153 patients with pain scores of 4 or higher in the Veterans Affairs primary care setting and used their progress notes to develop an information extraction schema on the quality of pain management documentation. Their schema identified three major areas—pain assessment, treatment, and reassessment—that covered several indicators such as cause, constancy, and pain sensation. In developing an annotation schema for anesthesia information and postsurgical pain, Yim et al [10] sampled 420 notes from patients that underwent five procedures. Many pain-related attributes aligned with those identified by Dorflinger et al [9] (eg, trigger, location, frequency, and pain character). Together, these studies confirmed that free-text notes contained relevant information for understanding pain and evidence of management approaches. Our study built upon this previous work and focused specifically on chronic pain. In particular, we developed an episode-based framework that allowed us to define each chronic pain entity longitudinally in a meaningful way.

## Methods

### Cohort Identification

Our study was approved by the Mayo Clinic and the Olmsted Medical Center (OMC) Institutional Review Boards (IRBs). The study population consisted of local adult patients with noncancer chronic pain receiving health care at the Mayo Clinic and/or the OMC. We used the Rochester Epidemiology Project (REP) [11,12] research infrastructure, which covers virtually all residents living in Olmsted County, Minnesota, between January 1, 2005, and September 30, 2015, totaling 189,475 persons. Patients who were coded with any *highly likely* code for chronic pain from the International Classification of Diseases, Ninth Revision (ICD-9), defined by Tian et al [7] (see Table 1), were included. Patients were excluded if they were younger than 19 years of age or if they had any ICD-9 code for cancer between 2003 and 2016; cancer was excluded because cancer-related pain has different treatment patterns. Accordingly, a cohort of 6586 patients out of 189,475 (3.48%) screened persons was established.

**Table 1:** International Classification of Diseases, Ninth Revision (ICD-9) codes determined by Tian et al [7] as *highly likely* to represent chronic pain.

ICD-9 code	Description
338.2	Chronic pain
338.21	Chronic pain due to trauma
338.22	Chronic post-thoracotomy pain
338.28	Other chronic postoperative pain
338.29	Other chronic pain
338.4	Chronic pain syndrome

**Figure 1:**
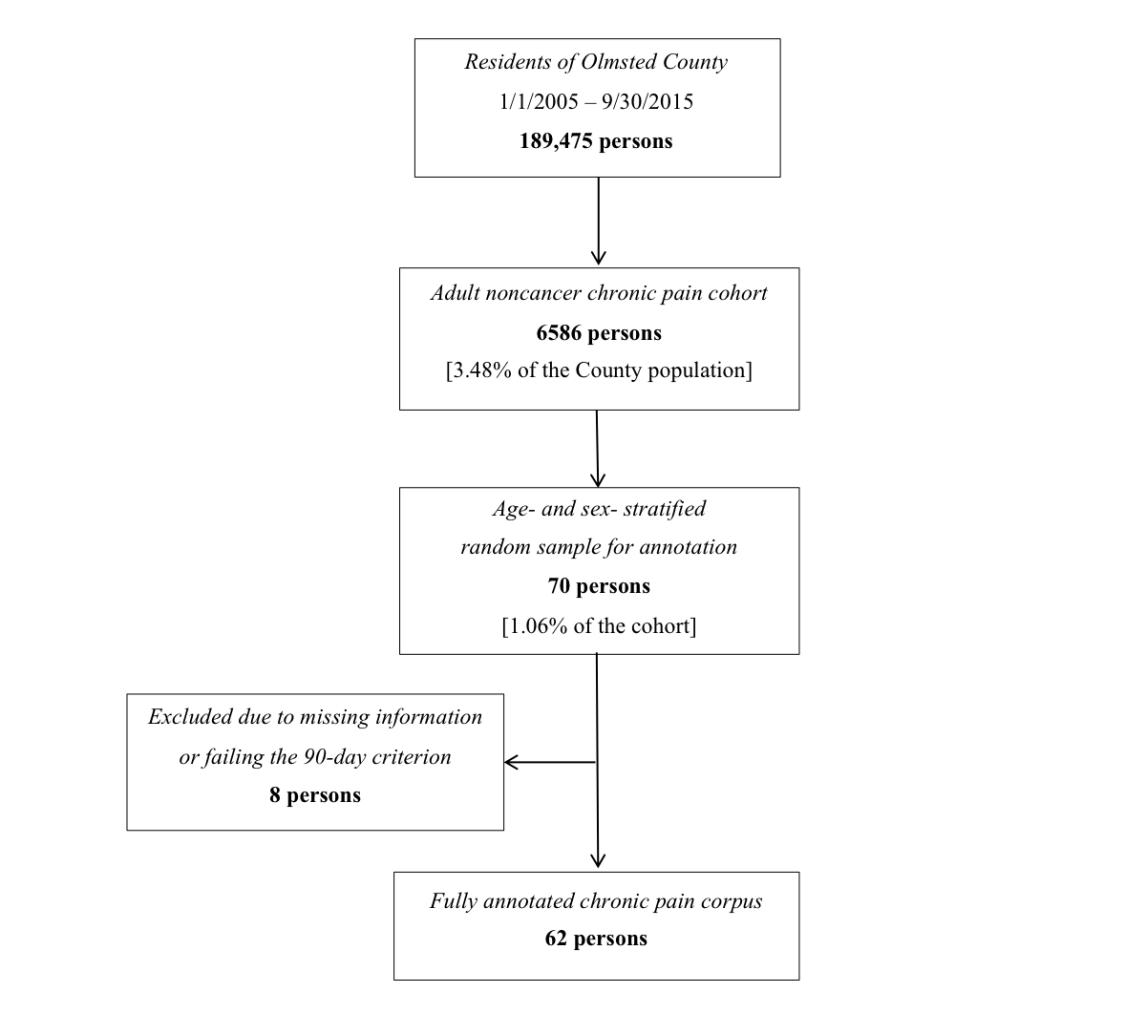
Workflow of screening the noncancer chronic pain cohort and sampling for the corpus annotation.

To diversify the demographics for more diverse and representative annotation, an age- and sex-stratified sample of 70 patients was randomly selected from the chronic pain cohort. The four age strata were 19-35, 36-50, 51-65, and >65 years of age. Of these 70 selected patients, 8 (11%) were later excluded from annotation due to the absence of any documented pain episode lasting 90 days or longer. An overview of the screening and sampling process is summarized in Figure 1.

### Pain Episode Framework

We proposed an episode-centered approach to annotating chronic pain based on input from clinical domain experts. Conceptually, each pain episode involved three points in time:

1. Initial event date: the date on which the pain first presented (eg, as the result of a fall). Since the initiation was not always specified in the EHR, the annotation could be an estimate and was not mandated for defining an episode.
2. Chronic pain index date: the date on which the pain was considered chronic (ie, at least 90 days after the initial event date). If the initial event date was unclear, then the first note implying that the pain had lasted at least 90 days or making explicit use of the word “chronic” was annotated as the chronic pain index date.
3. Last mention date: the date on which the pain was considered to be resolved or the patient was censored. This date could be determined by a note explicitly indicating resolution of the pain, no further mentions of the pain after that note, or a cutoff at 2 years after the index diagnosis if neither of the aforementioned criteria were met.

**Figure 2:**
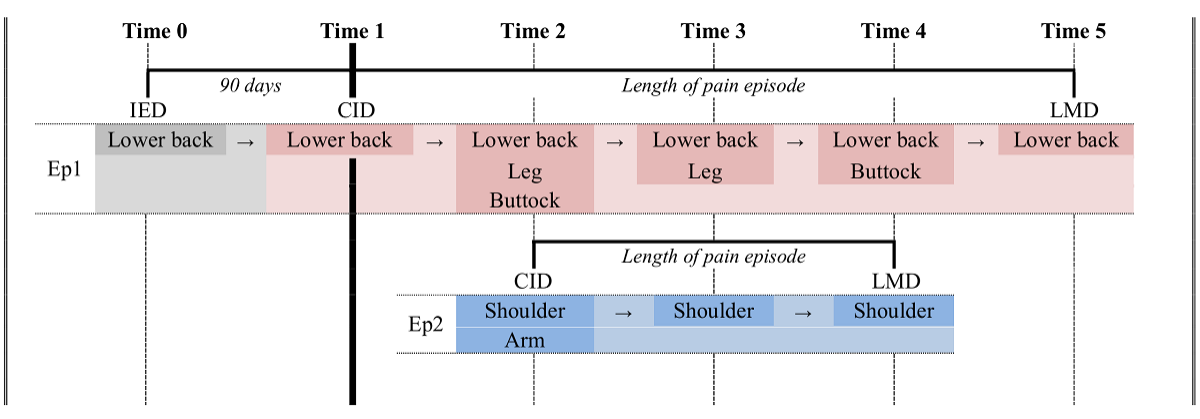
Conceptual representation of a patient’s two chronic pain episodes that unfolded in parallel. Note that the even time intervals are a simplified illustration; the real events have varying intervals. CID: chronic pain index date; Ep1: episode 1; Ep2: episode 2; IED: initial event date; LMD: last mention date.

Operationally, a chronic pain episode was defined by the chronic pain index date and last mention date plus at least one consistent location mentioned over time. If multiple locations were mentioned in the clinical note, annotators used their judgment to determine whether the locations could be physiologically linked. When the locations were all generated from one source (eg, lower back pain and leg pain due to sciatica), all locations were annotated as part of the same episode. As an example, Time 2 in Figure 2 illustrates how five pain locations can be annotated into two separate episodes, where episode 1 had started and evolved in parallel with the later episode 2.

### Corpus Annotation and Analysis

The first chronic pain ICD-9 code from structured data served as the anchor for corpus preparation, however, this diagnosis date might be different from the chronic pain index date determined later by an annotator; all of the patient’s clinical notes 6 months before and 2 years after this anchor diagnosis were then retrieved for annotation. The Multi-document Annotation Environment [13] was the annotation tool of choice because it is lightweight and easy to set up. The primary annotation tasks were (1) screening each patient’s notes to verify that they had at least one pain episode lasting 90 days or longer, (2) determining the pain episode boundary by identification of the chronic pain index date, last mention date, and initial event date, if applicable, and (3) marking mentions of the pain and associated attributes including date, location, severity, cause, effects on life, diagnostic procedure, medication, and other treatment regimens.

During the initial guideline development phase, multiple iterations of revisions were performed on both the guideline and the annotations. Each clinical note was independently annotated by two annotators (LC and MJ), and the interannotator agreement (IAA) was evaluated using the F1 score [14]. After each iteration, disagreements were resolved through discussion with a third reviewer (WH or JS). The common disagreements were logged and analyzed. The annotation guideline stabilized after going through such dual annotation and reconciliation over 604 notes. Following the guideline development, a total of 3272 notes representing 62 patients were reviewed by the two annotators in parallel. Upon completing the corpus annotation, descriptive statistics were computed to summarize the chronic pain episodes and attributes.

### Ethics Statement

The research involved secondary use of health records and did not involve a clinical trial. Because the research only used data passively collected as part of clinical care, and did not involve patient contact, the protocol was categorized as minimal risk. The requirement for informed consent was waived by the governing IRBs (Mayo Clinic: 18-006536 and Olmsted Medical Center: 038-OMC-18). We note that while informed consent was not required, Minnesota state law requires that health care providers collect and maintain patient authorization for linking medical record information across health care providers for research. All health care providers participating in the REP maintain research authorization, and we did not include the medical record information for patients who declined research authorization (<5% of potential participants).

### Data Availability

Deidentified annotations of the chronic pain episodes are available for noncommercial research purposes. Interested parties can request access by contacting rstnlp@mayo.edu and are required to sign and remain compliant with a Data Use Agreement under the Mayo Clinic NLP (natural language processing) Program (IRB 20-001137).

## Results

### Summary of the Annotated Cohort

**Figure 3:**
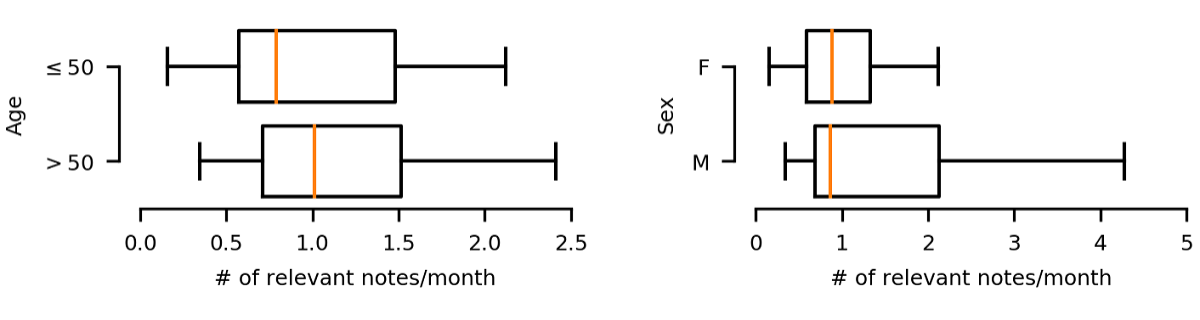
Boxplots for the per-month distributions of the annotated clinical notes, stratified by age (left) and by sex (right). Orange lines indicate medians; boxes are based on interquartile range. F: female; M: male.

The final annotated cohort consisted of 62 patients (34/62, 55% female) with balanced representation (approximately 15 patients) from each of the four age strata. To assess the density of relevant documentation, we computed the frequency distributions of the chronic pain–annotated notes per month (see Figure 3). Aligning with intuition, older patients (>50 years) had more clinical notes per month that documented chronic pain. The medians of relevant notes per month for men and women were comparable, but men exhibited a wider variance toward the higher end.

**Figure 4:**
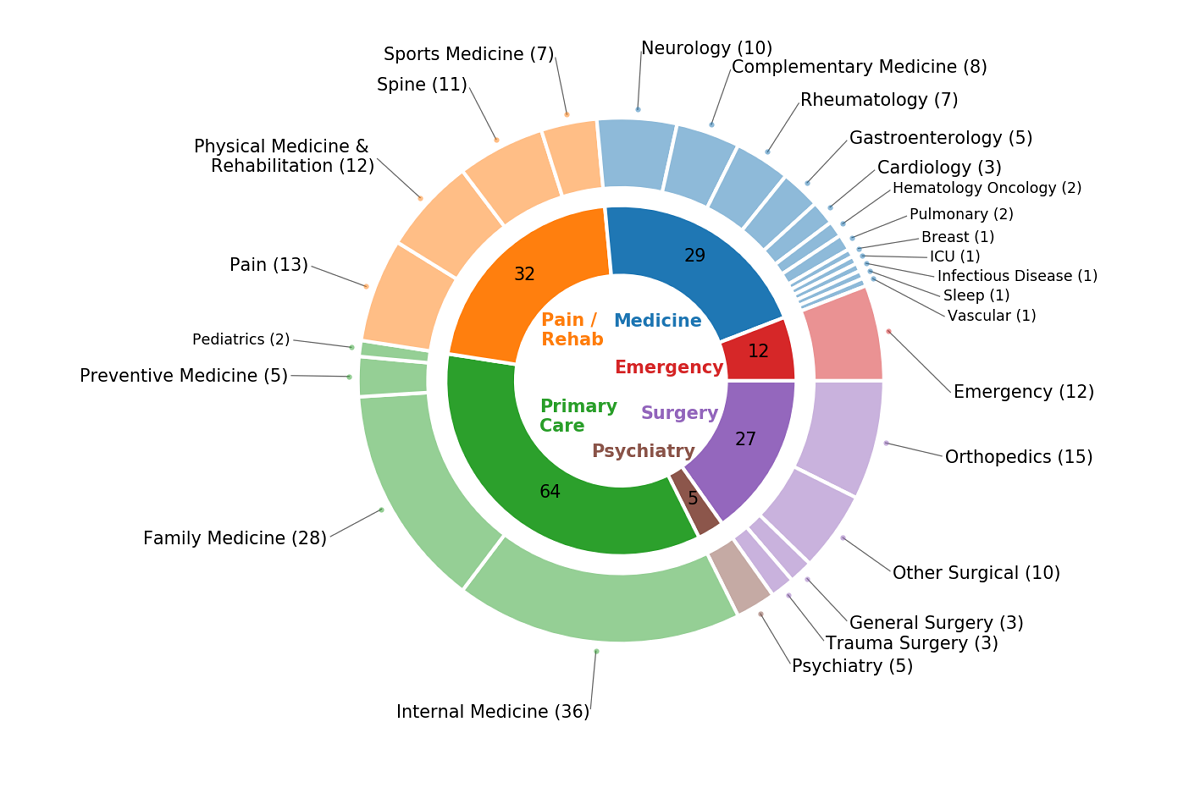
Counts of the annotated pain episodes by specialty. The inner circle represents the broader specialty category; the outer circle hosts the individual specialties under each category. Each quantity simply indicates how many episodes out of the 94 were cared for by the corresponding specialty or category. Note that an episode could be cared for by more than one specialty, so the counts involve overlaps and the sums may not match across. ICU: intensive care unit; Rehab: rehabilitation.

A total of 94 chronic pain episodes were identified from the 62 annotated patients (see Table 2). The median duration of each pain episode was 357 days (min 90 and max 977). To describe which specialties contributed to caring for and documenting the chronic pain patients within this cohort, we computed the specialty-wise episode counts in Figure 4. Primary care departments led the coverage (64/94, 68%), followed by pain and rehabilitation specialties (32/94, 34%) and a variety of medical specialties (29/94, 31%).

**Table 2:** Frequency distribution of patients per number of annotated chronic pain episodes.

Episodes, n	Patients (N=62), n (%)^a^
1	40 (65)
2	14 (23)
3	5 (8)
4	2 (3)
5	1 (2)

^a^Percentages do not add up to 100% due to rounding.

### Summary of the Annotated Corpus

In the guideline development phase, 30 episodes from 604 clinical notes were dually annotated. The IAA rates at the episode and concept levels are provided in Table 3. Note that the concept-level IAA overpenalized because each annotator had the freedom to extract the same information from any legitimate piece of evidence, for the same episode, but those differentially located pieces were counted as mismatches. As our primary focus, the episode-level IAA indicated moderate to high agreement (0.78 to 1.00) and viability of the final annotation guideline (see Multimedia Appendix 1).

**Table 3:** Interannotator agreement (IAA) rates computed using the F1 score.

Annotation	Episode-level IAA rate	Concept-level IAA rate
Episode	0.89	0.79
Date	1.00	0.94
Location	0.84	0.82
Severity	0.78	0.70
Cause	0.90	0.68
Social and emotional effect	0.78	0.53
Diagnostic procedure	0.78	0.65
Medication	0.82	0.70
Other treatment	0.80	0.65

**Figure 5:**
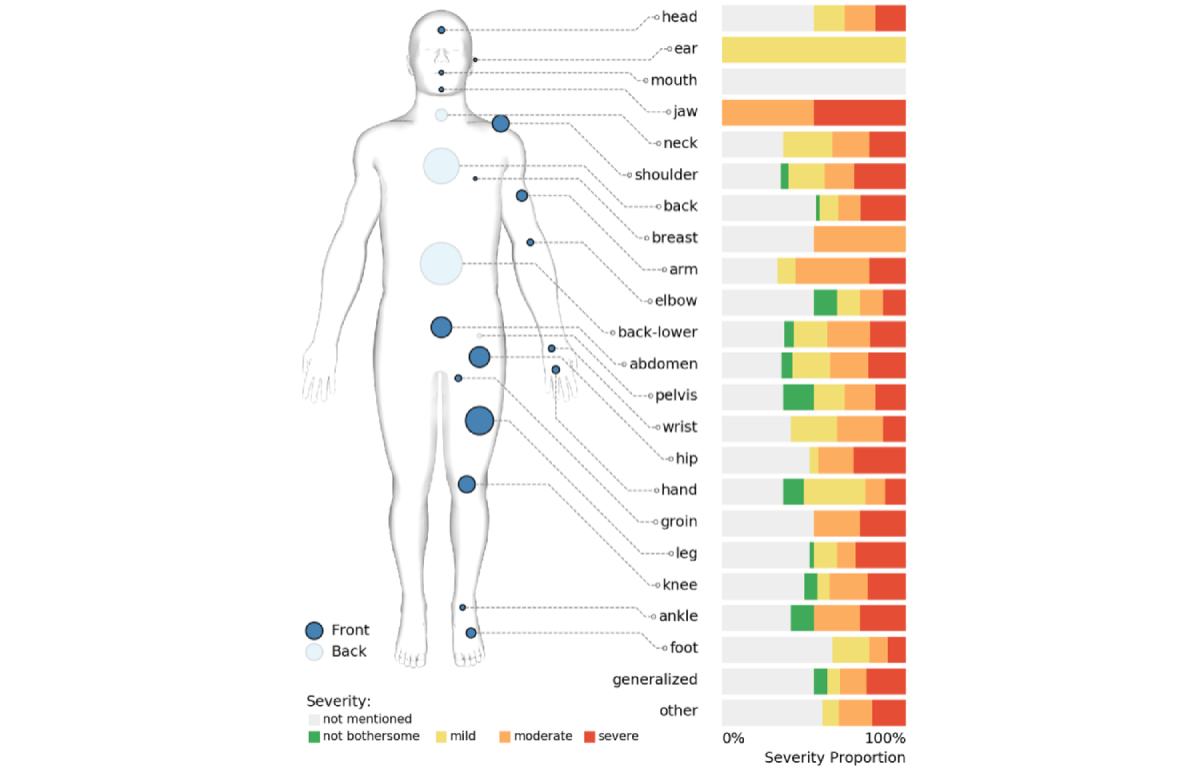
The annotated chronic pain episodes, grouped by body location and with corresponding severity distributions. The sizes of the blue circles on the figure reflect the relative frequencies within the annotated cohort.

**Figure 6:**
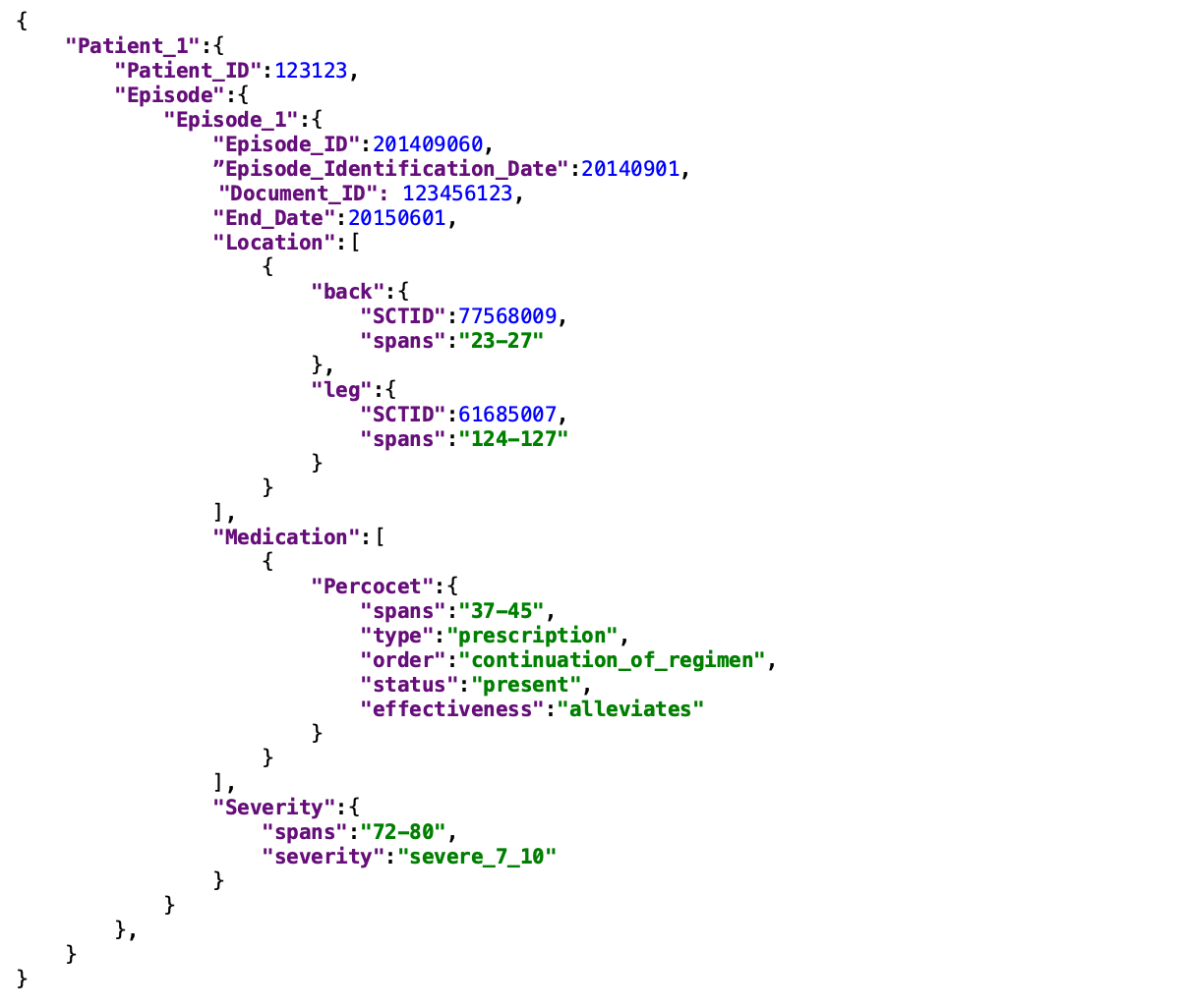
Illustration of an annotated episode exported into JavaScript Object Notation (JSON) format. Here, the mock-up patient had one episode of severe chronic pain in the back and the leg and received Percocet, which alleviated the pain. SCTID: SNOMED CT (Systematized Nomenclature Of Medicine–Clinical Terms) Identifier.

The individual pain attributes and corresponding examples are summarized in Table 4, along with the percentage of episodes that had the attribute covered. For example, 100% (94/94) of the episodes had *date* annotated, while only 59% (55/94) described the *social and emotional effect* of pain. Example annotations and the number of distinct strings of each annotated attribute are also included in the table. The most frequent subcategories of each annotated attribute are provided in Multimedia Appendix 2 (for *cause*, *social and emotional effect*, *diagnostic procedure*, *medication*, and *other treatment*) and Figure 5 (for *location* and *severity*). Structured export of a mock-up episode is shown in Figure 6, which represents the commonly included information: patient ID; episode begin and end dates; location, with mapping to SNOMED CT (Systematized Nomenclature Of Medicine–Clinical Terms); severity; medication; document ID; and the character spans of the annotations in text.

**Table 4:** Chronic pain attributes, examples, distinct strings, and the frequency of episodes that had the attribute annotated.

Attribute	Definition	Examples of annotation	Distinct strings, n	Coverage of episodes (N=94), n (%)
Date	Chronic pain index date, last mention date, or initial event date	“05-23-2009” “January 12, 2010”	174	94 (100)
Location	Body part where the pain occurred	“left knee” “lower back”	240	94 (100)
Severity	Perceived pain intensity	“tolerable” “9/10”	295	79 (84)
Cause	Etiology or contributing factor	“arthritis” “peripheral neuropathy”	390	88 (94)
Social and emotional effect	Consequence to daily life	“unable to bathe” “wakes him up at night”	152	55 (59)
Diagnostic procedure	Procedure used in investigating the pain	“chest x-ray” “bloodwork”	268	70 (74)
Medication	Pharmacological pain treatment	“Oxycodone” “Tylenol”	593	77 (82)
Other treatment	Nonpharmacological methods used to alleviate the pain	“cortisol injection” “suggested CBT” (cognitive behavioral therapy)	789	83 (88)

The pain *location* and *severity* information are presented jointly in Figure 5. Back pain, including lower back pain, and leg pain were the most common types of chronic pain in this annotated cohort. Pain severity distributions varied by body location, but statistical testing was not performed due to the limited sample size for each body location.

*Cause* and *social and emotional effect* were two critical aspects of chronic pain that we sought to elicit from clinical text, as they are generally not available through structured data. Musculoskeletal issues were the leading cause of pain in the annotated episodes (61/94, 65%) (eg, degenerative arthritis or patellar tendinitis). Note that it was possible for a pain episode to have more than one cause (ie, mechanism). For example, an episode of musculoskeletal lower back pain could also be annotated as neuropathic if it included sciatica. The diverse social and emotional pain effects manifested both diminished efficacy and compromised quality of life.

...[the pain] wakes him up at night.

...affect his ability to study and perform lab work for schooling.

...has missed a couple of family functions due to pain.

We were also interested in the management aspects of chronic pain documented in the clinical notes. Opioids were found to be the most frequently used *medication* (49/94, 52%), followed by nonsteroidal anti-inflammatory drugs and acetaminophen. Physical and occupational therapies were the most frequently documented nonpharmacological *other treatment* (41/94, 44%), followed by analgesic injections and surgery. Another valuable aspect revealed in the narratives was sentiment around the treatments, as shown below. We did not attempt to assign numeric ratings to these sentiments, as sentiment analysis was not a major focus of this study.

...has been trying Tylenol without much relief.

...is wishing to discontinue her Cymbalta.

...consider gradually tapering down the Neurontin.

### Interrater Disagreements

Most disagreements resulted from asymmetric annotation presence (ie, one annotator identified something the other annotator did not). In resolving these disagreements, we determined that some represented underannotation and others overannotation.

#### Underspecified Concept Definition

Asymmetric presence of annotations usually emerged due to unclear or inadequate extensional definition of the attribute to be annotated. The typical scenario was that an annotator did not realize that an entity should be annotated. For example, “discomfort at rest” can be annotated as mild *severity*, but such qualification was not apparent unless specifically named in the guidelines. The inconsistency could be rooted in differential interpretation or domain literacy, which were compensated by the iteratively refined guidelines through patching of inclusion criteria as annotators gained more experience.

#### Overinference of Evidence

One annotator tended to extrapolate evidence beyond the text, which resulted in many disagreements on *location* and *social and emotional effect*. A typical example was that “carpal tunnel syndromes” got annotated as *wrist*. Although *wrist* could be inferred as the pain location, our discussion stipulated that the annotators should stick to the literal description (ie, syndrome instead of anatomic location) without overinference. This criterion was incorporated into the guidelines to discourage inferring evidence that is not explicitly mentioned.

### Guideline Evolution

A summary of guideline evolution over the course of the study is available in Multimedia Appendix 1, section II, subsection 4. As the guideline was developed and used, some attributes were refined to make annotations more informative. For example, subcategories such as injury and trauma were added to the *cause* attribute to make the selections better fit the data. Other attributes were dropped due to scarce mentions in the corpus. Examples of dropped attributes include pain *trend*, which was intended to summarize whether pain was increasing, decreasing, or staying the same, and *referral,* which would identify a clinician referring a patient to another service.

## Discussion

### Principal Findings

Our episode-centered approach aligns closely with how clinicians identify and manage chronic pain: as an evolving and dynamic process. In some cases, including for aspects as central as the *cause* of chronic pain, clinicians exhibited changing attribution over time in their documentation, reflecting updated hypotheses as new information emerged (eg, from diagnostic interventions, progression of symptoms, or response to treatments). These time-varying considerations exemplify the complexity of accurately characterizing a chronic pain episode, which is more nuanced and multifaceted than simple concept extraction. Determining the episode boundaries can be nontrivial even for human annotators and relies on often imperfect or incomplete information in the text. It is also important to verify which pieces of evidence are credible and up to date whenever discrepancies in documentation are found. These challenges demand innovative solutions from the informatics community.

This study corroborated our hypothesis that rich information about chronic pain is available in clinical text and can be extracted with rigorous and standardized annotation. Some observations agreed with previous data, which have noted that back pain [15] was one of the most common causes of chronic pain, and opioids were a leading treatment choice in chronic pain [16]. Our annotation also produced novel data. For example, we found that chronic pain management is a multidisciplinary team effort that engages multiple medical and surgical departments, suggesting that optimal management will require active coordination that is attentive to specialty contexts.

Being the first clinical corpus dedicated to chronic pain, our experience could be considered prototypical with much room for improvement. Nonetheless, we believe the annotation guideline offers a solid starting point that can be referenced by other institutions with shared interest in abstracting chronic pain episodes from EHRs. We did not intend primarily to create or validate a corpus for machine learning, but we leave it to the potential users to determine how they want to leverage it. As a seed data set representing chronic pain annotations based on two health organizations, the corpus should benefit future work that has either a clinical or technical concentration (see Data Availability section for access information).

### Limitations

To maximize the number of individual patients we could analyze, given limited annotator time, we prospectively decided to analyze notes from 6 months before to 2 years after the index diagnosis date. A distribution analysis in Table 5 finds that only 8 of the 94 (9%) episodes were censored at 24 months. As a result of this design decision and our stratified sampling method, our data should not be used to estimate average chronic pain episode length in a broader population.

Although our results summarize more than 3000 clinical notes, the sample size of 62 patients is not large. The studied cohort—patients from Midwestern United States with high access to health care—may not fully represent other populations within the United States. However, we believe many of our findings are representative of chronic pain and may serve as a useful comparison for populations with very different characteristics [17]. Moreover, the study population was derived from two health care systems, representing both academic and community practices. It is important to note that the ICD-9 codes used in this study may have excluded chronic pain of many other possible causes. Finally, as in any study using human annotators, the work reflected the knowledge and possibly subjective understanding of those who performed the task.

Future work can address these limitations by testing reproducibility in a different cohort and with an expanded cohort definition and time period. Another promising path we envision is to reconstruct a richly characterized trajectory for each chronic pain episode by interweaving pertinent evidence from multiple types of data.

**Table 5:** Distribution of episode-ending distance from the index diagnosis date.

Month after the index diagnosis date when episode ended	Episodes (N=94) , n (%)
0	21 (22)
2	3 (3)
4	5 (5)
6	3 (3)
8	5 (5)
10	3 (3)
12	5 (5)
14	1 (1)
16	9 (10)
18	5 (5)
20	14 (15)
22	12 (13)
24	8 (9)

### Conclusions

To assess information about chronic pain in EHR notes, we performed systematic manual annotation on an age- and sex-stratified cohort of patients receiving health care at two health care systems between 2005 and 2015. An annotation guideline was iteratively refined to target key information about chronic pain episodes and associated attributes, such as *cause*, *location*, *severity*, and *medication*. A total of 3272 clinical notes were reviewed, and 94 episodes from 62 distinct patients were annotated. Summary statistics and qualitative analysis yielded insight into the characteristics of the cohort and their experiences. Clinical text was found to contain critical evidence for understanding the chronic pain trajectories of the patients. The episode-centered extraction captured a natural view of chronic pain while posing new challenges to potential automation.

## Appendix

Multimedia Appendix 1 Guidelines on annotating chronic pain in clinical text.

Multimedia Appendix 2 Subcategories of the annotated extrinsic chronic pain attributes.

## Abbreviations

EHR: electronic health record
IAA: interannotator agreement
ICD-9: International Classification of Diseases, Ninth Revision
IRB: Institutional Review Board
NLP: natural language processing
OMC: Olmsted Medical Center
REP: Rochester Epidemiology Project
SNOMED CT: Systematized Nomenclature Of Medicine–Clinical Terms
