# Supplementary material for: Characterizing Chronic Pain Episodes in Clinical Text at Two Health Care Systems: Comprehensive Annotation and Corpus Analysis
Source: JMIR Med Inform. 2020 Nov 16;8(11):e18659. doi: 10.2196/18659 (PMC7704279; doi:10.2196/18659)
Supplement: Multimedia Appendix 1 [file medinform_v8i11e18659_app1.pdf]

Guidelines on Annotating Chronic Pain in Clinical Text

Revision 4

5/13/2019

I Guidelines

1 Background

Unstructured EHR clinical notes contain a wealth of information on the diagnosis, treatment, and prognosis of persons with non-cancer, chronic pain conditions. However, it is time-consuming and costly to manually extract information from clinical records for large patient populations. To address this problem, several groups of investigators, including our own, have used NLP algorithms to identify clinical conditions and biomedical concepts from radiology reports, discharge summaries, problem lists, nursing documentation, and medical education documents. In this study, we will mine EHRs using NLP techniques to identify the presence of chronic pain among persons with either diagnostic codes likely to indicate chronic pain or persons with long term use of opioid medications. We will also extract information on pain severity and duration, types of treatment, and treatment effectiveness. Our goal is to develop, benchmark, and evaluate NLP algorithms to identify characteristics of chronic pain patients from unstructured Mayo Clinic clinical notes (Mayo Clinic EHR).

Our study population consists of persons who were residents of Olmsted County, MN between 1/1/2005 and 12/31/2016, and did not have a diagnosis of cancer between 1/1/2003 and 12/31/2016. We then further narrowed the cohort to 189,475 persons who were residents between 1/1/2005 and 09/30/2015 to avoid changes in codes between ICD-9 and ICD-10. Our final study cohort consisted of 6,586 adults (aged 19+) with a "highly likely" chronic pain code (Tian et al., 2013).

2 Annotation Tool

The annotation tool for this project is the Multi-document Annotation Environment (MAE), a Java-based natural language annotation software package. MAE is a non-web-based annotation tool (https://github.com/keighrim/mae-annotation). All annotation tasks will be defined in a document type definition (DTD) file. Due to its lightweight feature, the software can be easily shared and updated across multiple sites without configuration and testing.

3 Instructions

Please use this document for annotation to make sure we are consistently collecting the same information in the same way.

Annotators will be given access to a sample of clinical notes with .txt file format. After opening a .txt file in the MAE tool and allowing MAE to convert the file to an .xml format, annotators will be tasked with two primary goals. First, annotators will verify whether or not the patient meets the criteria for "chronic pain" (ref. 4.1 below). Second, annotators will identify and highlight keywords or phrases pertaining to the following concepts: date, location, severity, cause, effect, diagnostic procedure, medication, and other regimen (see **Table A.1** for details).

Annotators will then create tags for the highlighted span of text by right clicking and selecting a concept from the drop-down menu (n.b. a given span of text can be tagged with more than one concept, e.g. "headache" could be tagged as Pain and Location). Once a tag is created, additional attributes can be selected from drop-down menus within the given tag menu (ref.

4.2 below). The annotation will end when all medical records of that patient for a 2-year period are reviewed.

Naming convention:
20150101_9999999_9999999_PHYSICAL.txt_0_INITIAL_ANY COMMENTS YOU LIKE.xml

Note id: add id for every note, regardless of whether there are zero, one, or multiple mentions of a pain instance.
Annotator's Initials
Comments: you can put anything except the underscore "_"

4 Definitions

4.1 Chronic Pain

Annotators will assign a definition of whether chronic pain is present or absent. We will use Tian et al. and Wolfe et al. for our definition of chronic pain: non-cancer pain that is continuous and persistent and lasting for more than 90 days.

4.2 Tag Names, Keywords/Phrases, and Attributes

Specifically, most keywords or phrases have multiple possible attributes, and each attribute has categories that must be selected.

Table A.1. Concept definitions, example, and attribute value sets.

Concept/Definition	Examples	Attributes	
Date<br>*critical dates related to genesis, identification, and resolving of pain*	"05-23-2009"<br>"January 12, 2010"	*date_type*	initial_event_date<br>chronic_pain_identified_date<br>last_mention_date
Location<br>*location of the pain in the body*	"left knee"<br>"lower back"<br>"proximal femur"	*location*	Abdomen (SCTID: 818983003)<br>Ankle (SCTID: 344001)<br>Arm (SCTID: 53120007)<br>back (SCTID: 77568009)<br>back-lower (SCTID: 264072009)<br>back-upper (SCTID: 264240001)<br>breast (SCTID: 76752008)<br>buttocks (SCTID: 110976001)<br>chest (SCTID: 51185008)<br>ear part (SCTID: 119262002)<br>elbow (SCTID: 127949000)<br>eye (SCTID: 81745001)<br>face (SCTID: 89545001)<br>foot (SCTID: 56459004)

			generalized (SCTID: 278001007)<br>groin (SCTID: 26893007)<br>hand (SCTID: 85562004)<br>head (SCTID: 69536005)<br>hip (SCTID: 29836001)<br>incisional<br>jaw (SCTID: 661005)<br>knee (SCTID: 72696002)<br>leg (SCTID: 61685007)<br>mouth (SCTID: 123851003)<br>neck (SCTID: 45048000)<br>nose (SCTID: 45206002)<br>pelvis (SCTID: 12921003)<br>shoulder (SCTID: 16982005)<br>throat (SCTID: 49928004)<br>wrist (SCTID: 8205005)<br>other
<u>Severity</u><br>*strength or intensity of the pain sensation*	"tolerable"<br>"9/10"	*attributes*	Not Bothersome (0) (SCTID: 81765008)<br>Mild (1-3) (SCTID: 40196000)<br>Moderate (4-6) (SCTID: 50415004)<br>Severe (7-10) (SCTID: 76948002)
<u>Cause</u><br>*etiology (if present) or likely factors contributing to the pain*	"fracture of the tibia"<br>"arthritis"<br>"peripheral neuropathy"	*caused_by*	injury_trauma (SCTID: 417746004)<br>surgical (SCTID: 257556004)<br>musculoskeletal (MSK) (SCTID: 279069000)<br>neuropathic (SCTID: 247398009)<br>non-MSK medical issue<br>indeterminate
<u>Effect</u><br>*effect of the pain on daily life/experience*	"missing a lot of school"<br>"annoyed that he is unable to bathe"<br>"wakes him up at night"	*manifest*	movement<br>sleep<br>work_or_school<br>social_family<br>other
		emotional	bothered<br>not_bothered
Diagnostic_procedure<br>*procedure performed to diagnose the cause of the pain*	"chest x-ray"<br>"bloodwork"	*status*	past<br>planned<br>requested<br>recommended<br>current
<u>Medication</u><br>*name of medications*<br>**relevant to pain treatment*	"Oxycodone"<br>"Tylenol"<br>"aspirin"	*type*	over_counter<br>prescription<br>herbal_natural_supplement<br>other

		order	new<br>change_in_dose<br>continuation_of_regimen<br>cancellation
		status	past<br>planned<br>requested<br>recommended<br>current
		effectiveness	alleviates<br>worsens<br>no_change
		side_effects	present<br>none
<u>Other_treatment</u><br>*non-pharmaceutical procedure/action to help alleviate pain*	“cortisol injection”<br>“referred to neurology”<br>“given educational materials about self-care”<br>“suggested CBT”	*type*	education<br>surgery<br>hospitalization<br>alternative_medicine<br>chiropractor<br>injection<br>PT_OT<br>other
		order	referral/consult<br>new<br>change_in_dose<br>continuation_of_regimen<br>cancellation
		status	past<br>planned<br>requested<br>recommended<br>current
		effectiveness	alleviates<br>worsens<br>no_change
		side_effects	present<br>none

1 Annotation Environment

Example with a mock-up note: annotation of date (CID in this case), location, cause (uncertain in this case), effects, previous medications and other regimens (highlighted), diagnostic interventions, and planned medications.

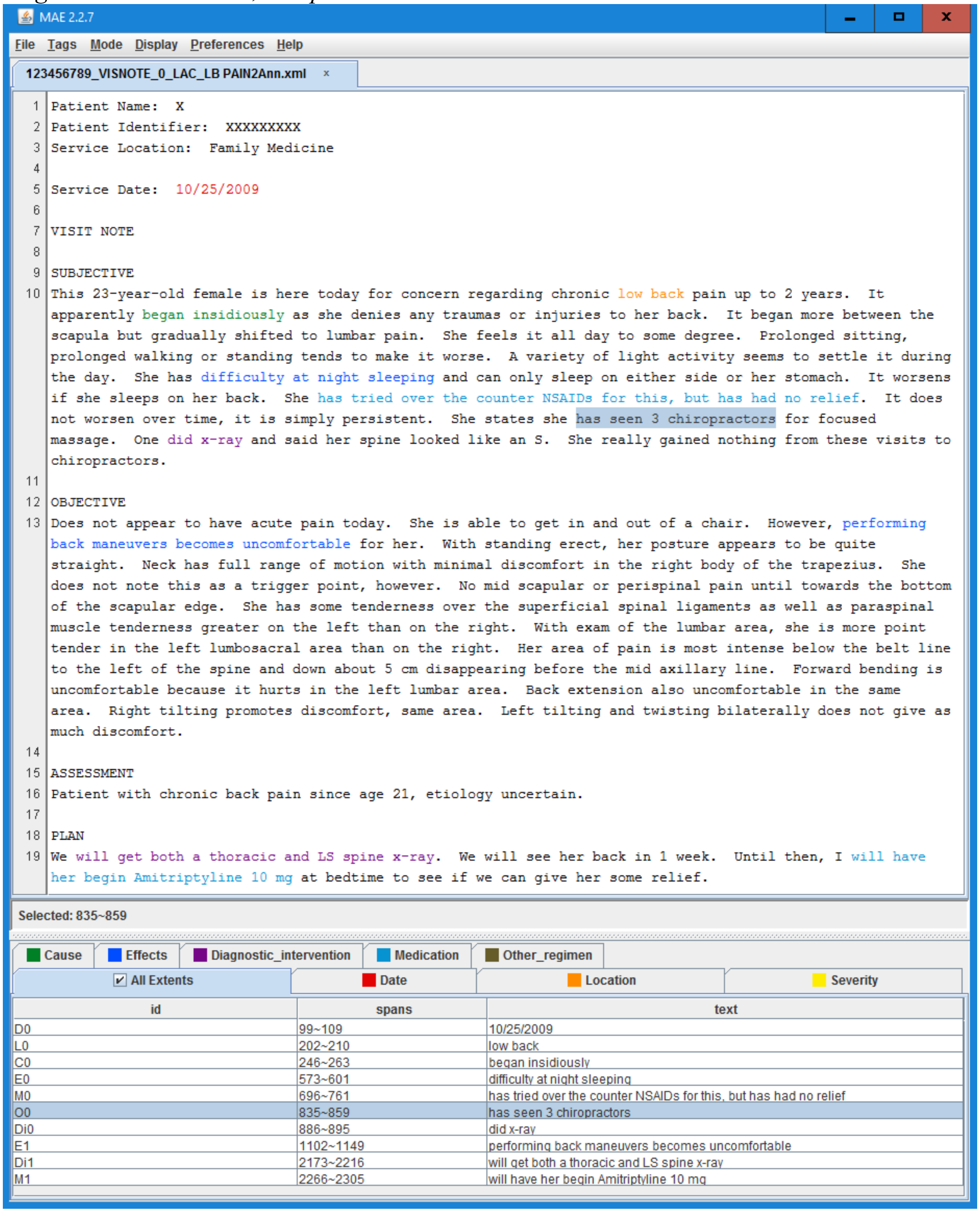

2. Specific annotation criteria

No Extrapolation of evidence:
Annotation should only be based on the textual information of the given context. Annotators should not make inference, assumptions or use any prior knowledge to make annotation decision. For example, after the annotators and adjudicator discussed, we decided not to annotate "carpel tunnel syndrome" with an additional anatomy concept of "wrist".

Mark semantically sufficient minimum span:
Annotators should treat each unique concept independently. Choose the smallest possible span that semantically enclose the problem, condition, or diagnosis, but do not chose a span that indicates something too generic or non-specific.

"John was jumping down off the tank when he jarred his back."
Cause

"John took a trial of Lyrica"
Medication

"John's pain intensity varies between a 2 to an 8 and limits his ability to do things"
Severity ***Effects***

In the first example, the main focus of this entry is 'jarred his back.' Do not include untreated expressions, such as 'jumping down off' as part of the focus. Similarly, 'a trial of' and 'pain intensity varies between' should not be annotated. Conversely, for the third example, do not chose simply 'ability' as the focus. Generally, the focus span should be as short as possible while still capturing the intent.

3. Examples of tricky cases

Location

Example	Annotator 1	Annotator 2	Adjudication
"Bilateral buttock and radiating leg pain in a sciatic-like fashion"	Definitely a location. It's tricky because it's two different locations	I would annotate with two separate tags	Highlight all, label two locations separately
"Carpal tunnel release"	Annotated as wrist	Issue with including "release" as part of the location	Don't annotate as location
"Carpal tunnel syndromes"	Annotated as wrist	Issue with including "syndrome" as part of the location. To me, "carpal tunnel" refers to the anatomical structure, whereas "carpal tunnel syndrome" refers to the painful condition. I realize "carpal tunnel" is often used as shorthand for the syndrome, but I feel it is necessary to draw the line somewhere in order to adequately capture the nuances of the semantics	Don't annotate as location

Medication

Example	Annotator 1	Annotator 2	Adjudication
"Attempting to decrease his pain medication"	I don't remember the context of this. The physician may not have written the name of the medication in the note. But it definitely refers to a change in dose of a pain medication	Lack of specificity, need to have medication name	Don't annotate as medication
"Continues to taper off his pain medication"	same as above.	Lack of specificity, need to have medication name	Don't annotate as medication

Social/emotional effect

Example	Annotator 1	Annotator 2	Adjudication
“He has been walking more often and is able to walk for longer periods”	implies improvement in effect=move	True, but are we looking to capture improvements that are not explicitly tied to some kind of intervention? My understanding of the "Effects" label is that it should only be applied to unambiguous instances of pain-affecting-experiencer type phrases. If anything, this phrase should be tagged with "Medication" or "Other treatments", but the reasons for improvement are not explicitly mentioned, so it kind of has to be left alone in my opinion.	Don’t annotate as effect
“He is eager to return to golf and riding his motorcycle.”	activities he is unable to complete=social effects	Maybe, although I'm not sure how I feel about the amount of inference required to glean "activities he is unable to complete" from "he is eager to return to (activities)". I certainly see that returning to an activity implies that one cannot currently participate in that activity, but this particular sentence is ambiguous as to why he is eager, e.g. is he eager because pain is limiting him, because some other issue is limiting him, or because he's just plain looking forward to returning to his activities? I'm on the fence about this one.	Don’t annotate as effect
“Previously very active with running, jogging, etc.”	implies he is no longer able to be active	Too much inference about previous activity level	Don’t annotate as effect
“Walking more often and is able to walk for longer periods”	implies improvement in effect=move	Not try to capture the improvement but the effects of the pain on the person's life	Don’t annotate as effect
“Will get her back into some hair cutting activities”	implies improvement in effect=work	Disagree--if I remember correctly, this phrase was in the context of some kind of compensation evaluation, so it was more of a declaration of intent on the part of the doctor (to allow the hairdresser back to work) than it was a comment on the decreased negative effects of the pain on her work.	Don’t annotate as effect

Severity

Example	Annotator 1	Annotator 2	Adjudication
“Enough discomfort in his back that sitting in a truck driving 7 hours a day would be very difficult for him”	Annotated as severity, but we can't apply mild/moderate/severe.	Although this is a rather long and complex way of conveying severity. Not sure if we want the "Severity" concept to encompass such narrative-styled descriptions.	Don’t annotate as severity
“Just a little bit tender over the left lower lumbar area”	Definitely describes severity	Not annotated as severity	Annotate as severity, label as mild
“Little in the way of neck pain”	Definitely describes severity	Not severity	Annotate as severity, label as mild
“Little or no neck pain”	Definitely describes severity	Not severity	Annotate as severity, label as mild
“No pain in his arm”	In context, this might have been to point out that the pain had resolved?	I wouldn't annotate as "Severity". If anything, this could be potentially used as part of "last mention date"	Annotate as severity, label as not bothersome
“Patient rates lowest level of pain at 6/10. Patient rates highest/worst level of pain at 9/10.”	Definitely describes severity; we don't have a way to split out lowest/worst pain	Should annotate separately	Annotate “pain at 6/10” as severity, label as moderate; annotate “pain at 9/10” as severity, label as severe
“Significant pain and discomfort”	Definitely describes severity	Not severity	Annotate as severity, label as severe
“Significant pain in his back”	Definitely describes severity	Not severity	Annotate as severity, label as severe

Other treatment

Example	Annotator 1	Annotator 2	Adjudication
“Much improvement with her right hand”	We don't have a place to put outcomes. We have to tie them to treatment. This was probably a note about physical therapy, so it was clear that the reference was to the effect of the PT.	Although it’s some kind of treatment related, it should not be annotated as treatment.	Don’t annotate as treatment

4. Guideline evolution
The initial annotation guideline was developed with the consultation of pain related literature (Yim et al. and Dorflinger et al.) and a Mayo Clinic physician specializing in chronic pain management. We followed an EHR-based consensus development process (Fu et al.) to evaluate the appropriateness of each chronic pain-related concept and corresponding annotations.

During iterative development process, we modified the annotation guidelines based on newly discovered evidence from the data. For example, the "chronic_pain_identified_date" was introduced for replacing "documentation date" in order to accurately capture the index date for each pain episode. We also decided to use SNOMED CT to normalize body locations. Based on the frequency of numerical and categorical values for severity, we created a grading schema to capture and normalize both data representations: 0 for Not Bothersome, 1-3 for Mild, 4-6 for Moderate, and 7-10 for Severe. We also modified the effect category to capture both physical and emotional components. The final annotation guideline was a synthesis of the initial guidelines and incremental findings from real world EHR. **Table B.2** gives a more detailed before/after comparison of the annotation schema. If the final version has additional contents is a row, it means the schema was expanded; if it is the opposite, it means certain attribute was dropped in the final version mainly due to few mentions observed in the corpus.

Table B.2. Comparison of the initial versus the final schema

**Initial version**	**Final version**
Index_date<br>• Date: incident_case | prevalent_case | unknown	Date<br>• Date: initial_event_date | chronic_pain_identified_date | last_mention_date
Cause	Cause<br>• Caused by: injury_trauma | surgical | musculoskeletal | neuropathic | other_medical | other
Diagnostic_intervention	Diagnostic_procedure<br>• Status: past | planned | requested | recommended | current
Location<br>• abdomen | ankle | arm | back | back-lower | back-upper | breast | buttocks | chest | ear | elbow | eye | foot | generalized | groin | hand | head | hip | incisional | jaw | knee | leg | mouth | neck | nose | pelvis | shoulder | throat | wrist | other	Location<br>• Location (SCTID): abdomen | ankle | arm | back | back-lower | back-upper | breast | buttocks | chest | ear | elbow | eye | face | foot | generalized | groin | hand | head | hip | incisional | jaw | knee | leg | mouth | neck | nose | pelvis | shoulder | throat | wrist | other
Severity	Severity<br>• Severity (SCTID): not_bothersome_0 | mild_1_3 | moderate_4_6 | severe_7_10

Trend<br>• increase | decrease | no_change	
Trigger<br>• increase | decrease	
Effect<br>• quality_of_life | resolved | other	Effect<br>• Manifest: resolved | move | sleep | work_or_school | social_family | other<br>• Emotional: bothered | not_bothered
Medication<br>• Type: over_counter | prescription | herbal_natural_supplement | other<br>• Order: new | change_in_dose | continuation_of_regimen | cancellation<br>• Status: past | planned | requested | recommended | current<br>• Effectiveness: alleviates | worsens | no_change<br>• Side_effects: present | none	Medication<br>• Type: over_counter | prescription | herbal_natural_supplement | other<br>• Order: new | change_in_dose | continuation_of_regimen | cancellation<br>• Status: past | planned | requested | recommended | current<br>• Effectiveness: alleviates | worsens | no_change<br>• Side_effects: present | none
Other_treatment<br>• Status: past | planned | requested | recommended | current<br>• Effectiveness: alleviates | worsens | no_change<br>• Side_effects: present | none)	Other_treatment<br>• Type: education | surgery | hospitalization | alternative_medicine | chiropractor | injection | PT_OT | other<br>• Order: referral_consult | new | change_in_dose | continuation_of_regimen | cancellation<br>• Status: past | planned | requested | recommended | current<br>• Effectiveness: alleviates | worsens | no_change<br>• Side_effects: present | none
Referral<br>• Status (past | planned | requested | recommended | current)	
Education<br>• Status (past | planned | requested | recommended | current)	

5. References/Links

1. Dorflinger et al, Development and application of an electronic health record information extraction tool to assess quality of pain management in primary care. TBM, 2014. 4:184-189.
2. Wolfe F, Smythe HA, Yunus MB, Bennett RM, Bombardier C, Goldenberg DL, Tugwell P, Campbell SM, Abeles M, Clark P, Fam AG. The American College of Rheumatology 1990 criteria for the classification of fibromyalgia. Arthritis & Rheumatism: Official Journal of the American College of Rheumatology. 1990 Feb;33(2):160-72.
3. Tian TY, Zlateva I, Anderson DR. Using electronic health records data to identify patients with chronic pain in a primary care setting. J Am Med Inform Assoc. 2013;20(e2):e275-280.
4. Yim et al, Annotation of pain and anesthesia events for surgery-related processes and outcomes extraction, Proceedings of the BioNLP 2017 workshop, pages 200-205, Vancouver, Canada, Aug 4, 2017
5. Leech G. Corpus Annotation Schemes. Literary and Linguistic Computing. 1993;8:275-81. 10.1093/llc/8.4.275.
6. Fu S, Leung LY, Raulli AO, Kallmes DF, Kinsman KA, Nelson KB, Clark MS, Luetmer PH, Kingsbury PR, Kent DM, Liu H. Assessment of the impact of EHR heterogeneity for clinical research through a case study of silent brain infarction. BMC medical informatics and decision making. 2020 Dec;20:1-2.
