# Supplementary material for: Characterizing Chronic Pain Episodes in Clinical Text at Two Health Care Systems: Comprehensive Annotation and Corpus Analysis
Source: JMIR Med Inform. 2020 Nov 16;8(11):e18659. doi: 10.2196/18659 (PMC7704279; doi:10.2196/18659)
Supplement: Multimedia Appendix 2 [file medinform_v8i11e18659_app2.pdf]

Summary of the Chronic Pain Annotation: Extrinsic Attributes

The following tables provide more detailed statistics and examples for the annotated extrinsic[§] attributes: chronic pain *cause*, *social/emotional effect*, *diagnostic procedure*, *medication*, and *other treatment*. The 'Episode Coverage' column contains the number of episodes and corresponding percentage (out of total 94 annotated episodes) that belong to the category of the row. The percentages may add up >100% because an episode can belong to multiple categories.

Table B.1. Most frequent pain causes in the annotated chronic pain episodes

**Cause**		
Category	*Episode Coverage*	*Examples*
MSK[a] issue	61 (65%)	"Left knee degenerative arthritis"<br>"patellar tendinitis"
Non-MSK[a] medical issue	27 (28%)	"mild form of celiac disease"<br>"abdominal migraine"
Indeterminate	24 (26%)	"Chronic pain syndrome"<br>"indeterminate etiology"
Injury or trauma	24 (26%)	"motor vehicle accident … 28 years ago"<br>"hurt his back"
Neuropathic origin	14 (15%)	"diabetic peripheral neuropathy"<br>"Fibromyalgia"
Surgical origin	3 (3%)	"Painful left total knee arthroplasty status post left knee revision"<br>"postoperative ... traumatic extraction"

[a]MSK = musculoskeletal

[§]For the distribution of intrinsic attributes (i.e., *location* and *severity*), please refer to Figure 5 in the article.

Table B.2. Most frequent social/emotional effects of chronic pain in the annotated episodes

**Social/emotional effect**		
Category	*Episode Coverage*	*Examples*
Sleep disturbance	31 (33%)	"says he cannot sleep"<br>"wakes him up at night"
Mobility	23 (25%)	'Functional limitations: posture and positioning"<br>"restricted elbow range of motion primarily due to pain"
Work or school	19 (20%)	"having difficulty even making it to work"<br>"affect his ability to study and perform lab work for schooling"
Other	18 (19%)	"problem eating rice ... gets bloating, pain and cramping"
Social or family	6 (6%)	"has been avoiding certain social situations"<br>"has missed a couple of family functions due to pain"

Table B.3. Most frequent diagnostic procedures in the annotated chronic pain episodes

**Diagnostic procedure**		
Category	*Episode Coverage*	*Examples*
X-ray	43 (46%)	"will go ahead and get a knee x-ray"
MRI[a]	37 (39%)	"scheduled an MRI scan of her hip tomorrow"
Labs	30 (32%)	"ordered some blood work"
EMG[b]	13 (14%)	"recently had an EMG"
Ultrasound	12 (13%)	"had a right upper quadrant ultrasound"
CT[c]	12 (13%)	"are obtaining a CT"

[a]MRI = magnetic resonance imaging

[b]EMG = electromyography

[c]CT = computed tomography

Table B.4. Most frequent medications in the annotated chronic pain episodes

Medication		
Category	*Episode Coverage*	*Examples*
Opioids	49 (52%)	"refilled tramadol"
NSAID[a]	31 (33%)	"naproxen"
Acetaminophen	23 (25%)	"trying Tylenol"
Antidepressants	19 (20%)	"Cymbalta"
Unclear	15 (16%)	"home pain medications"
GABA[b] analogs	12 (13%)	"Neurontin"

[a]NSAID = nonsteroidal anti-inflammatory drug

[b]GABA = gamma-Aminobutyric acid

Table B.5. Most frequent other (non-pharmacological) treatments in the annotated chronic pain episodes

Other treatment		
Category	*Episode Coverage*	*Examples*
PT[a] or OT[b]	41 (44%)	"may benefit from a physical therapy program"
Analgesic injections	26 (28%)	"only received benefit from several injections"
Surgery	23 (24%)	"After pyriformis release, she feels that she is doing better"
Education	9 (10%)	"Patient education materials provided"
Hospitalization	6 (6%)	"hospitalized for acute chronic abd pain"

[a]PT = physical therapy

[b]OT = occupational therapy
